# Mr. Rolfe's Case of Small-Pox

**Published:** 1801-07

**Authors:** W. D. Rolfe

**Affiliations:** Dispensary House, North Street


### *To the Editors of the Medical and Phyſical Journal.*

GENTLEMEN,

MR. HEBB's caſe of Small-pox induced me to ſtate the following caſe, as deviating a little from the general courſe of ſymptoms, and if thought worthy a place in your Journal, you will oblige me by inſerting it.

I was ſent for at ſix in the morning to a woman in labour, who was delivered at nine; ſhe had ſlight pains the day before, and a trifling head-ache, but no other indiſpoſition, and that ſo ſlight as not to be noticed by her friends. I called on her the next morning; ſhe was hot and thirſty, which I attributed to the ſmall room in which ſhe lay, being very cloſe, and a fire in it: Her pulſe was natural, and her tongue moiſt; ſhe expreſſed herſelf comfortably. On the ſame evening, (Friday) 11 o'clock, I was ſent for, and was informed, ſhe had a raſh out on her; I told them directly it looked like the Small-pox, the mother ſaid, it could not be that, as ſhe had it when twelve years old, and was marked with it in the face, which I could not then diſcover. On calling the next morning I was fully convinced it was the Small-pox; and I ſuppoſe the mother, or her friends, had been miſtaken reſpecting the former diſeaſe: She had the preſent very full, but did well.

As ſoon as the child was born it was taken from the mother and carried away, (as it was not intended by the friends to be ſuckled by the mother); on the eighth day the child was brought home with the Small-pox, out very full; it became confluent, and the child ſoon died. The child was never put to the breaſt, and was taken away before the eruption appeared on the mother.

I am, &c.

*Dispensary House, North Street,* W. D. ROLFE.

